# Evaluation of Saponin-Rich Callus from *Saponaria officinalis* L. as a Novel Scrub Material with Significant Exfoliating and Anti-Inflammatory Effects

**DOI:** 10.3390/plants14101535

**Published:** 2025-05-20

**Authors:** Ga-Ram Yu, Da-Hoon Kim, Hyuck Kim, Dong-Woo Lim

**Affiliations:** 1 Department of Diagnostics, College of Korean Medicine, Dongguk University, Goyang-si 10326, Republic of Korea; kalama2@dongguk.edu; 2 Institute of Korean Medicine, Dongguk University, Goyang-si 10326, Republic of Korea; 3 TOPO Lab., Co., Ltd., Goyang 10326, Republic of Korea; kimdahoon@topo.co.kr (D.-H.K.); hk_ceo@topo.co.kr (H.K.)

**Keywords:** *Saponaria officinalis* L., plant callus, cosmetic ingredient, novel scrub material, anti-inflammatory effect, exfoliating effect, clinical skin test

## Abstract

*Saponaria officinalis* L., a plant rich in saponins, has long been used as a natural surfactant. It has traditionally been used for its cleansing and anti-inflammatory properties in the treatment of various skin conditions, including eczema, psoriasis, and acne. In this study, we investigated the potential of *S. officinalis* callus (SC), mass-produced via plant tissue culture, as a novel exfoliating cosmetic ingredient. The callus was induced using Murashige and Skoog (MS) medium supplemented with 1 mg/L 2,4-D, and the resulting extract (SCE) was analyzed via high-performance liquid chromatography (HPLC), confirming the presence of saponarin—a bioactive compound with known anti-inflammatory properties. In vitro assays demonstrated that SCE significantly suppressed nitric oxide production and reduced the expression of pro-inflammatory mediators, including iNOS, COX-2, TNF-α, IL-1β, and IL-6, in LPS-stimulated RAW264.7 macrophages. The foaming ability and stability of SC and SCE were also comparable to commercial surfactants. Clinical studies further supported the material’s cosmetic potential: a skin patch test in 30 volunteers revealed no signs of irritation (mean score: 0.28), while a desquamation index assessment in 21 participants showed a significant reduction of 44.07%, confirming its exfoliating efficacy. Taken together, these results suggest that the SC scrub is a safe, eco-friendly, and sustainable alternative to synthetic exfoliating agents, offering functional and industrial advantages for cosmetic applications.

## 1. Introduction

The epidermis consists of five distinct layers, with the stratum corneum (SCO)—the outermost layer composed of corneocytes—being the terminally differentiated form of keratinocytes [1]. Skin turnover, a process by which aged epidermal cells are gradually pushed toward the surface to form the SCO, is essential for maintaining both the aesthetic appearance and overall health of the skin [2,3]. Various internal factors such as aging, moisture loss, and lipid depletion, as well as external factors including ultraviolet (UV) exposure and environmental stressors, affect the integrity and function of the SCO [4]. Excessive retention of the SCO can lead to skin concerns such as dullness, acne, and dryness [5,6].

The SCO plays a pivotal role in maintaining the skin barrier by preventing transepidermal water loss (TEWL) and acting as the first line of defense against environmental insults, including microbial invasion, allergens, and chemical irritants [7]. Disruption of this barrier—due to either excessive accumulation of corneocytes or impaired desquamation—can trigger cutaneous inflammation and subsequently activate innate immune responses [8].

Conversely, inflammatory stimuli originating from within the skin can disrupt epidermal homeostasis. Pro-inflammatory cytokines such as TNF-α, IL-1β, and IL-6 have been shown to stimulate keratinocyte hyperproliferation [9] and alter differentiation patterns, leading to abnormal thickening of the SCO [10]. These mechanisms are commonly observed in chronic inflammatory skin conditions such as psoriasis and atopic dermatitis [11]. Therefore, addressing inflammation-related changes in the epidermis, along with maintaining proper exfoliation, is essential to preserve skin health and prevent disease progression.

Skin exfoliation, which can be categorized into mechanical and chemical methods, refers to the removal of dead cells from the skin surface to improve texture, appearance, and overall skin condition [12]. To prevent and delay skin aging, scrubs enriched with bioactive molecules that eliminate impurities and cleanse deeply are considered an effective strategy [13]. Plastic microbeads have traditionally been used as exfoliants; however, due to their negative environmental impact, especially aquatic pollution, they are being gradually phased out [14]. In response, the cosmetics industry is increasingly seeking sustainable alternatives to non-degradable polymers that can offer comparable exfoliating performance [15]. Naturally derived exfoliants such as plant seeds, natural fibers, salts, and sugars are emerging as viable replacements [16,17].

In most major families of terrestrial plants, wounded tissues regenerate through the formation of undifferentiated callus cells [18]. When cultivated in vitro, plant callus cells can serve as efficient and competitive systems for the production of biologically active and marketable secondary metabolites [19]. Notably, treating these undifferentiated cells with elicitors can enhance the biosynthesis of such metabolites [20]. Additionally, plant calluses can be induced to form either friable or compact types, depending on the culture conditions, which determine their texture and hardness—properties advantageous for various industrial applications [21,22]. In our previous study, we reported the favorable properties of *Cannabis sativa* callus extract as a cosmetic ingredient, which exhibited both anti-inflammatory and antioxidant activities [23]. Based on these findings, we suggested that plant calluses and their extracts could serve as sustainable and competitive novel cosmetic materials, and that other plant species may hold similar potential for industrial use [23].

*Saponaria officinalis* L. (commonly known as “soapwort”) is a perennial herb in the Caryophyllaceae family, native to Europe [24]. It produces high levels of glycosidic compounds, known as saponins, which can reduce surface tension to levels comparable to those of synthetic surfactants [25]. Traditionally, *S. officinalis* has been used for its cleansing and anti-inflammatory effects in the treatment of skin conditions such as eczema, psoriasis, and acne [26]. The plant contains various saponins, such as quillaic acid, gypsogenin, and hederagenin, as well as flavonoid compounds like saponarin [27]. Among these, saponarin is known for its anti-diabetic, antioxidant, anti-inflammatory, and anti-allergic properties [28,29]. Moreover, *S. officinalis* possesses a notably high saponin content and well-characterized surfactant properties, which are considered superior to those of many other plant species [30]. Combined with its rapid growth, ease of propagation, and demonstrated anti-inflammatory effects, these features make it a highly attractive and practical candidate for further development as a functional natural ingredient. Given these attributes, we hypothesized that the SC could serve as a novel cosmetic material, particularly as a natural exfoliant with added anti-inflammatory benefits.

In this study, we investigated the potential of *S. officinalis* callus (SC) and its extract (SCE), both rich in saponins, as a new scrub material through a series of experiments. Our findings demonstrated that SCE exhibited notable anti-inflammatory activity, potentially mediated by its major component, saponarin. Additionally, the SC and SCE showed favorable foaming ability and stability. Clinical skin tests revealed no safety concerns and confirmed the exfoliating efficacy of SC. Collectively, these results support the potential of SC as a safe, effective, and sustainable exfoliating agent with promising industrial applicability.

## 2. Results

### 2.1. Effect of Saponaria officinalis L. Callus Extract (SCE) on RAW264.7 Cell Viability

**Figure 1:**
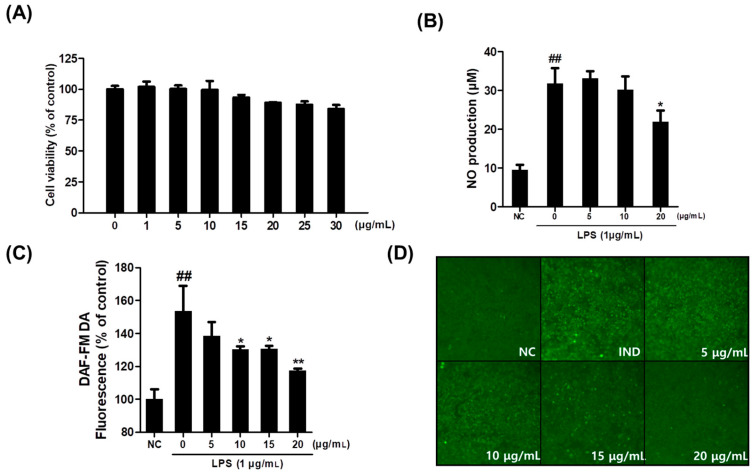
Effect of callus extract on RAW264.7 cell viability and NO production. (**A**) RAW264.7 cells were incubated for 24 h with *Saponaria officinalis* L. callus extract (SCE, 0–30 μg/mL). Results are expressed in percentages of untreated controls. (**B**,**C**) The extracellular NO levels were measured using the Griess reagent, and intracellular NO concentrations were measured using the DAF-FM DA assay. (**D**) Fluorescence images of cells showing NO production levels based on signal intensities. NC, normal control; IND, lipopolysaccharide (1 μg/mL); SCE, *S. officinalis* L. callus extract (0–20 μg/mL) + LPS. The results are presented as the means ± SDs of three independent experiments. ^##^ *p* < 0.01 versus untreated controls, and * *p* < 0.05 and ** *p* < 0.01 versus LPS-treated RAW264.7 cells.

RAW264.7 cells were incubated with callus extracts at various concentrations (1, 5, 10, 15, 20, 25, and 30 μg/mL) for 24 h. A significant decrease in cell viability was observed at 30 μg/mL (83.9%), while no biologically significant cytotoxicity was detected at 20 μg/mL (88.87%). A widely accepted and rigorous threshold for determining cytotoxicity and the criteria for assessing cytotoxic effects often vary depending on the specific study design and experimental context [31,32,33]. Based on these results, considering these findings together with our experimental result showing a cell viability of 88.87%, subsequent experiments were performed using concentrations lower than 20 μg/mL (Figure 1A).

### 2.2. Effect of Saponaria officinalis L. Callus Extract (SCE) on Intracellular or Extracellular NO Levels

RAW264.7 cells were treated with SCE in the presence of 1 μg/mL lipopolysaccharide (LPS). The level of nitric oxide (NO) released into the culture medium was measured using the Griess reaction. Treatment with 20 μg/mL of SCE significantly reduced LPS-induced NO production from 31.84 μM to 21.84 μM (Figure 1B). Additionally, intracellular nitrite levels were assessed using the DAF-FM DA assay. SCE treatment markedly reduced fluorescence intensity at an excitation/emission wavelength of 485/535 nm, indicating decreased intracellular NO levels (Figure 1C). DAF-FM-DA staining was visualized in RAW264.7 cells. As shown in Figure 1D, intracellular NO production increased in the LPS-only treatment group, and the increased intracellular NO production induced by LPS was dose-dependently reduced in fluorescence intensity by SCE treatment (Figure 1D).

### 2.3. Effect of Saponaria officinalis L. Callus Extract (SCE) on the Inflammatory Mediator Levels in LPS-Stimulated RAW264.7 Cells

**Figure 2:**
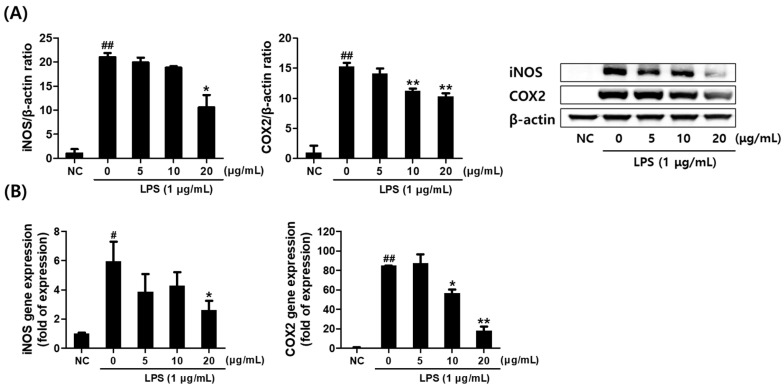
*Saponaria officinalis* L. callus extract (SCE) inhibited LPS-induced up-regulations of iNOS and COX2 in RAW264.7 cells. (**A**) Relative protein levels of iNOS and COX2 were determined by Western blot and their representative blot images are presented. (**B**) Relative gene expression levels of iNOS and COX2 as determined by real-time PCR. The results are presented as the means ± SDs of three independent experiments. ^#^ *p* < 0.05 and ^##^ *p* < 0.01 versus untreated controls and * *p* < 0.05 and ** *p* < 0.01 versus LPS-treated RAW264.7 cells.

The effects of SCE on the expression of inflammation-related mediators were evaluated by examining the mRNA and protein levels of inducible nitric oxide synthase (iNOS) and cyclooxygenase-2 (COX-2) in LPS-stimulated RAW264.7 cells. Treatment with SCE resulted in a dose-dependent reduction in iNOS protein expression, consistent with the observed decrease in NO production, showing statistical significance at 20 μg/mL. Similarly, COX-2 protein levels were significantly reduced at concentrations of 10–20 μg/mL (Figure 2A). Furthermore, SCE dose-dependently suppressed both the mRNA and protein expression of iNOS and COX-2 in a pattern consistent with the protein-level observations (Figure 2B).

### 2.4. Effect of Saponaria officinalis L. Callus Extract (SCE) on the Pro-Inflammatory Cytokine Levels in LPS-Stimulated RAW264.7 Cells

**Figure 3:**
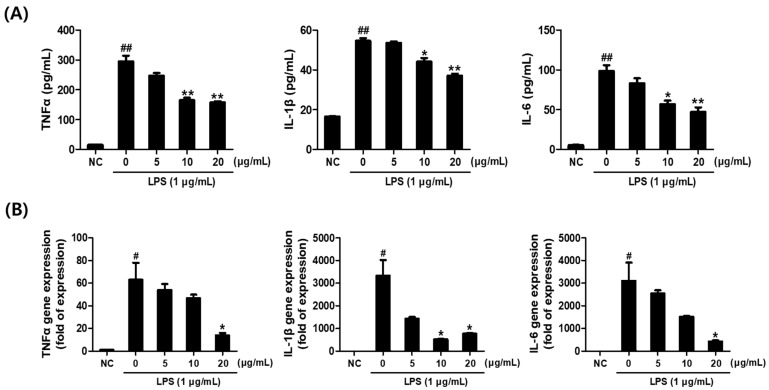
*Saponaria officinalis* L. callus extract (SCE) reduced LPS-induced increases in pro-inflammatory cytokine levels in RAW264.7 cells. (**A**) Relative expression of TNF-α, IL-1β, and IL-6 as determined by ELISA. (**B**) Relative expression of TNF-α, IL-1β, and IL-6 as determined by qPCR. The results are presented as the means ± SDs of three independent experiments. ^#^ *p* < 0.05 and ^##^ *p* < 0.01 versus untreated controls and * *p* < 0.05 and ** *p* < 0.01, versus LPS-treated cells.

To evaluate the anti-inflammatory potential of SCE, ELISA and quantitative PCR (qPCR) were performed to measure LPS-induced cytokine production. Supernatants collected from cultured RAW264.7 cells were analyzed for TNF-α, IL-1β, and IL-6 using commercial ELISA kits. Co-treatment with SCE significantly and dose-dependently inhibited the LPS-induced secretion of TNF-α, IL-1β, and IL-6 proteins (Figure 3A). Consistently, qPCR analysis revealed that SCE markedly downregulated the mRNA expression levels of these pro-inflammatory cytokines, with statistical significance observed at 20 μg/mL (Figure 3B).

### 2.5. Profiling and Identification of Major Compounds in Callus Extract by High-Performance Liquid Chromatography (HPLC)

**Figure 4:**
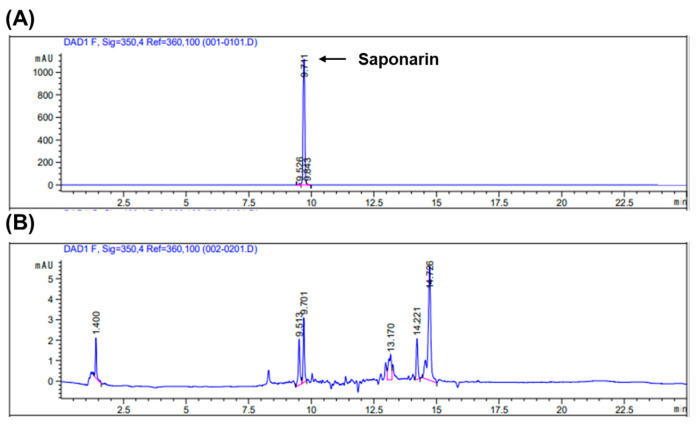
HPLC fingerprinting analysis of *Saponaria officinalis* L. callus extract (SCE)**.** (**A**) HPLC chromatogram of saponarin, a known standard compound of *Saponaria officinalis* L. (**B**) HPLC chromatogram of SCE.

High-performance liquid chromatography (HPLC) fingerprinting analysis was conducted on SCE to identify major constituents, with a focus on saponarin—a known compound in *Saponaria officinalis* L. The chromatographic profiles of commercial saponarin (5 mg/mL) and the callus extract (10 mg/mL) are shown in Figure 4. The retention time of the saponarin standard was 9.711 min (Figure 4A), while a major peak in the SCE was observed at 9.701 min, suggesting the presence of saponarin. Based on peak area comparison, the concentration of saponarin in the callus extract was estimated to be 10.49 μg/mL (Figure 4B).

### 2.6. Foaming Ability and Stability Test of Callus Extract and Scrub by Bartsch Test

**Figure 5:**
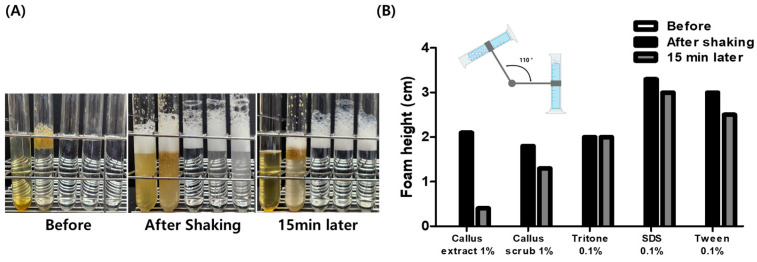
In vitro foaming ability and stability test using *Saponaria officinalis* L. callus (SC) and its extract (SCE). Maximum foam height generated was measured immediately after shaking, and foam decay was measured after 15 min of shaking. (**A**) The image shows the height of foam generated, and (**B**) the height of the generated foam was measured and quantified.

Foaming ability and stability were evaluated using the Bartsch test, in which samples were placed in measuring cylinders and shaken simultaneously to compare foam height. Immediately after shaking, both the callus extract and the scrub exhibited comparable foaming ability to 0.1% Triton X-100. However, after 15 min, the SC demonstrated superior foam stability and maintained a higher foam height compared to the SCE (Figure 5).

### 2.7. Clinical Tests of Saponaria officinalis L. Callus (SC) Scrub for Skin Irritability and Exfoliatiory Effect

To evaluate the safety of the SC scrub on human skin, a patch test was conducted with 30 healthy volunteers. Participant information is provided in Supplementary Table S1. The average skin irritation score was 0.28, indicating no signs of irritation and demonstrating the product’s skin safety.

**Figure 6:**
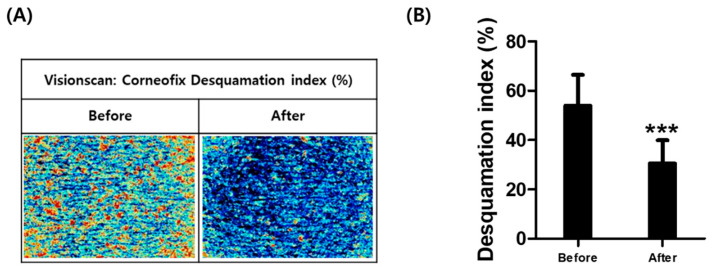
Corneofix desquamation index analysis using Visioscan. (**A**) Representative Visioscan images from a test volunteer (No: P013) showing a visible reduction in dead skin cells before and after SC scrub application. (**B**) Average desquamation index (%) from 21 volunteers. *** *p* < 0.001, compared to baseline (before scrub use).

The exfoliating efficacy of the SC scrub was assessed in 21 volunteers (Supplementary Table S2). Visioscan imaging revealed a visible improvement in corneofix desquamation after product use (Figure 6A). Quantitative analysis showed a statistically significant 44.07% reduction in the skin desquamation index following SC scrub application (Figure 6B), suggesting effective removal of dead skin cells and confirming its exfoliating function.

**Figure 7:**
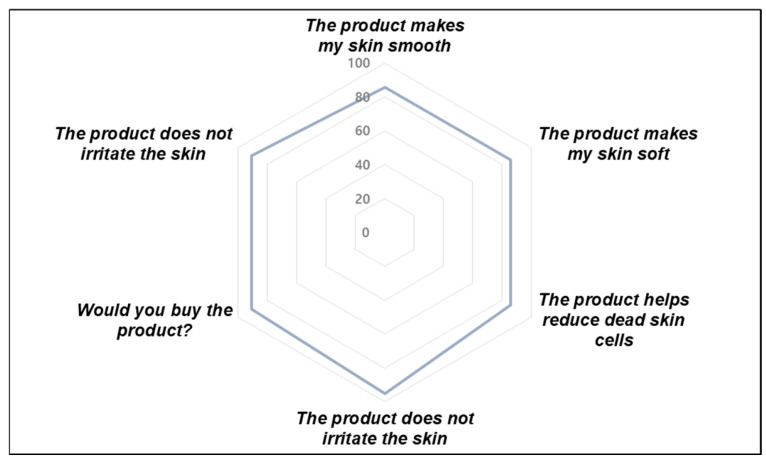
Self-assessment survey results from test volunteers (n = 21) following the use of *Saponaria officinalis* callus (SC) scrub. The graph shows the percentage of participants who reported a significantly positive response (“Satisfied” or “Very satisfied”) to each of the six statements in the self-assessment questionnaire, evaluated after the first use of the product.

In a self-assessment survey completed by participants, 85.71–90.47% of participants provided positive feedback on six aspects of the SC scrub, including its effectiveness and distinct sensory attributes, and expressed a willingness to purchase the product (Figure 7 and Supplementary Table S3).

## 3. Discussion

The demand for functional cosmetic products and biologically active ingredients has steadily increased in recent years. In the cosmetics industry, there is a growing interest in innovative and differentiated raw materials, particularly those derived from natural sources with proven efficacy and safety. Additionally, as awareness of the environmental and health concerns associated with microplastics in cosmetic scrubs increases, the search for natural exfoliating alternatives has gained significant momentum [34]. These newly introduced natural exfoliants must satisfy multiple criteria, including low production costs, solvent resistance, mechanical durability, smooth surface morphology, particle size uniformity, and strong efficacy and safety profiles [35].

In this study, callus from *Saponaria officinalis* L. (SC)—a saponin-rich plant—and its extract (SCE) were investigated as a potential natural scrub material. Plant-derived ingredients that can be mass-produced through sustainable technologies such as plant tissue culture are highly valued due to their environmental sustainability and industrial applicability. Our previous study has reported that SCE possess significant antioxidant and immunomodulatory properties [36]. Furthermore, one study showed that SCE inhibited the production of inflammatory mediators in LPS-induced macrophages and zebrafish models, effects attributed to its major component, saponarin, which was proposed to act through interaction with the TLR/MyD88 signaling pathway [30,37]. In this context, SC offers a novel exfoliating alternative that differs fundamentally from conventional seed- or mineral-based scrubs in terms of origin, composition, and functional profile.

Keratinocytes, beyond serving as structural components of the epidermis, also act as immune modulators by secreting various inflammatory cytokines and mediators, including TNF-α, IL-6, IL-1α, IL-10, and granulocyte–macrophage colony-stimulating factor [38,39]. Additionally, the NO produced during inflammatory responses plays a regulatory role in inflammation and stimulates the proliferation and re-epithelialization of endothelial cells [40,41]. The differentiation of keratinocytes is regulated by cytokines, which are crucial for maintaining the skin barrier. In particular, an imbalance in pro-inflammatory cytokines can disrupt keratinization and impair barrier function [10]. By modulating these inflammatory mediators, SCE—an active ingredient in the SC scrub—may offer therapeutic benefits for skin conditions associated with inflammation and impaired epidermal homeostasis.

Our clinical results reinforce the potential of SC as a cosmetic ingredient. In a skin irritation test involving 30 volunteers, SC scrub exhibited no signs of irritation, with a mean irritation index of 0.28—indicating excellent skin compatibility, even for sensitive users. Furthermore, in an exfoliation efficacy test with 21 participants, the desquamation index was significantly reduced by 44.07%, demonstrating a strong exfoliating effect comparable to or exceeding that of commercially available natural scrubs. The freeze-dried SC showed suitable physical characteristics (hardness and size stability at room temperature), making it an ideal candidate for use in scrub formulations. Additionally, the saponin content may facilitate the removal of dead skin cells while minimizing irritation.

Surfactants are essential components in cosmetic and personal care products due to their roles in emulsification, foaming, wetting, dispersion, and cleansing. Some surfactants also exhibit additional antioxidant and antimicrobial properties [42]. However, anionic surfactants have been shown to rapidly increase skin conductivity and water permeability, leading to nonlinear increases in skin penetration. Surfactants with higher micelle charge densities have caused more significant skin damage, including corneocyte swelling, lipid layer disruption, and enhanced permeability to ions, water, and the surfactant itself [43].

In addition, synthetic surfactants may negatively impact skin health by disrupting the resident microbiome community [44]. The skin is colonized by diverse commensal microorganisms, which play a beneficial role by preventing the growth of pathogenic species [45]. However, certain cosmetic products or ingredients can disturb this microbial balance, potentially contributing to skin disorders and immune dysregulation [46,47]. In contrast, plant-derived surfactants such as saponins offer several advantages over synthetic, petroleum-based surfactants, including lower toxicity, biodegradability, and environmental friendliness [48]. As a result, natural surfactants of plant origin are actively being explored as sustainable alternatives in cosmetic formulations worldwide [49,50].

Consistent with previous reports highlighting *S. officinalis* as a rich source of natural surfactants, both the SCE and SC scrub exhibited excellent foam-generating capacity, as demonstrated by the Bartsch test. These results have important industrial implications, suggesting that callus-derived materials retain key bioactive properties of the parent plant. Furthermore, the in vitro callus culture system provides an efficient and sustainable platform for the continuous production of natural saponins, with advantages in cost reduction, time efficiency, and scalability.

However, pharmacological effects may vary between plant extracts and their corresponding callus extracts due to differences in chemical composition [51]. Therefore, further studies are needed to evaluate whether the SCE displays biological activity equivalent to that of the whole plant extract. Additionally, several considerations must be addressed for commercialization: achieving particle uniformity, validating formulation stability, and conducting long-term safety and efficacy assessments.

Given these strengths and remaining challenges, SC represents a promising natural exfoliant with multifaceted benefits for skin health. Its anti-inflammatory, cleansing, and exfoliating properties, combined with high biocompatibility and eco-friendliness, position it as an attractive ingredient for next-generation functional skincare products.

In conclusion, with its demonstrated efficacy and proposed mechanism of action, a cosmetic formulation containing SCE may offer significant anti-inflammatory benefits. Moreover, the SC scrub shows strong potential as a natural exfoliating agent that effectively removes dead skin cells without causing irritation, supporting its application in functional cosmetic development.

## 4. Materials and Methods

### 4.1. Chemicals

Murashige and Skoog (MS) callus induction medium, 2,4-dichlorophenoxyacetic acid (2,4-D) and plant agar were purchased from Duchefa (Haarlem, The Netherlands). Dulbecco’s Modified Eagle’s Medium (DMEM) was obtained from Hyclone (Logan, UT, USA), and fetal bovine serum (FBS) and penicillin–streptomycin solution were purchased from Invitrogen (Carlsbad, CA, USA). LPS and other reagents were obtained from Sigma Aldrich (St. Louis, MO, USA). Primary antibodies against iNOS, COX-2, β-actin, Lamin B, and secondary antibodies were obtained from Santa Cruz (Dallas, TX, USA). The ELISA kits for mouse TNF-α, IL-1β, and IL-6 were supplied by R&D Systems (Minneapolis, MN, USA). The oligonucleotide primers used for real-time qPCR were supplied by Macrogen (Seoul, Republic of Korea).

### 4.2. Seed Sterilization and Callus Induction

Seeds of *Saponaria officinalis* L. were purchased from Natural Farm (Gangwon-do, Republic of Korea). The seeds were sterilized in 70% ethanol for 2.5 min, followed by sterilization in a 12% sodium hypochlorite solution for 25 min. The seeds were rinsed thoroughly with sterile distilled water to remove residual disinfectants, peeled, and placed on callus induction medium. The cultures were incubated for 30 days at 28 °C under a 16 h light/8 h dark photoperiod with a light intensity of 142 μmol/m^2^/s (PPFD).

### 4.3. Preparation of Callus Induction Medium

**Figure 8:**
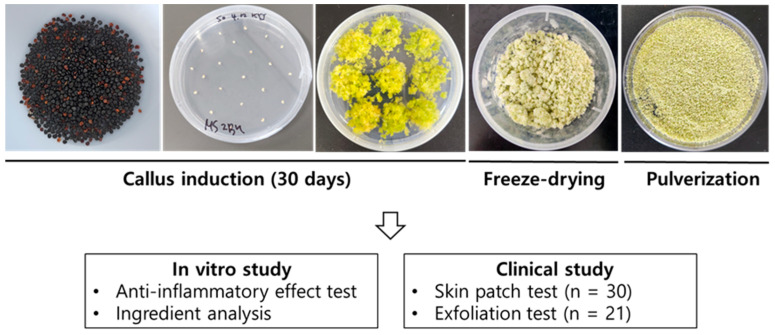
Illustration demonstrating the procedure of this study. *Saponaria officinalis* callus (SC) induction, processing of calluses, bioactivity evaluation of SC extract (SCE) in vitro, and clinical test results using SC scrub.

To induce callus formation in *Saponaria officinalis* L., 3% sucrose (30 g/L), 4.66 g of M.S medium, and 0.8% plant agar (8 g) were precisely measured and added to a 1 L glass bottle. After adding 1 L of water, the pH was adjusted to 5.6–5.8 using 1 N NaOH or 1 N HCl solution. The pH-adjusted medium was then sterilized in an auto-clave at 120 °C for 15 min. After cooling, 2,4-D was added to final concentrations of 1 mg/L. The medium was then dispensed into Petri dishes (25 mL per dish) and allowed to solidify at room temperature. The whole process of callus induction is illustrated in Figure 8.

### 4.4. Preparation of Callus Scrub and Extract

After harvesting and freeze-drying the callus, the material was pulverized into a fine powder. The resulting powder was used directly as the scrub material. For extract preparation, 20 g of the callus powder was extracted in 100 mL of 50% ethanol at room temperature for 72 h. The ethanol was then removed using a rotary evaporator (Buchi, Switzerland), and the residue was freeze-dried to yield 8.9 mg of powdered extract. The obtained extract was dissolved in dimethyl sulfoxide (DMSO) at a concentration of 100 μg/mL and filtered through a 0.22 μm syringe filter prior to use in subsequent experiments (Figure 8).

### 4.5. Cell Culture and Treatment

RAW264.7 cells, a murine macrophage cell line, were obtained from the Korea Cell Line Bank (KCLB, Seoul, Republic of Korea). Cells were cultured in DMEM supplemented with 10% FBS and 1% penicillin–streptomycin at 37 °C in a humidified atmosphere containing 5% CO_2_. To evaluate the anti-inflammatory effects of the callus extract (SCE), cells were co-treated with LPS (1 μg/mL) and SCE for 6 h (for real-time PCR and immunofluorescence microscopy) or for 12–24 h (for nitrite measurement, Western blotting, and ELISA).

### 4.6. Cell Viability Assay

Cell viability was assessed using the EZ-Cytox assay kit (Daeil Lab, Seoul, Republic of Korea) according to the manufacturer’s instructions. RAW264.7 cells were seeded in 96-well plates at a density of 3 × 10^3^ cells per well and incubated overnight at 37 °C in a 5% CO_2_ incubator. The following day, cells were treated with various concentrations (1, 5, 10, 15, 20, 25, and 30 μg/mL) of SCE and incubated for 24 h under the same conditions. After replacing the culture medium with FBS-free DMEM, EZ-Cytox reagent was added, and cells were incubated for an additional 30 min. Optical density (OD) was measured at 450 nm using a microplate spectrophotometer (VersaMax, Molecular Devices, San Jose, CA, USA).

### 4.7. Measurement of Extracellular and Intracellular NO Levels

The extracellular production of NO was measured using the Griess reagent system. RAW264.7 cells (3 × 10^4^ cells/mL) were seeded in six-well plates and incubated for 24 h. Cells were then co-treated with LPS (1 μg/mL) and various concentrations (5, 10, and 20 μg/mL) of the callus extract for an additional 24 h. The nitrite concentration in the culture medium, as an indicator of NO production, was determined by mixing the medium with Griess reagent, and the OD was measured at 540 nm using a microplate spectrophotometer. Nitrite concentrations were calculated based on a standard curve generated using sodium nitrite.

For intracellular NO detection, RAW264.7 cells were seeded in black 96-well plates at a density of 3 × 10^3^ cells/well. After 24 h, cells were co-treated with LPS (1 μg/mL) and various concentrations (5, 10, 15, and 20 μg/mL) of callus extract for 24 h. Cells were then incubated with DAF-FM DA (5 μM) and washed three times with Dulbecco’s phosphate-buffered saline (DPBS). Fluorescence intensity was measured using a fluorescence microplate reader (Spectra Gemini, Molecular Devices, San Jose, CA, USA) at excitation/emission wavelengths of 485/545 nm. Representative fluorescence images were captured using an Olympus BX50 fluorescence microscope (Olympus, Tokyo, Japan).

### 4.8. Western Blotting

The expression levels of inflammation-related proteins were analyzed using Western blotting. Briefly, cells were lysed in radioimmunoprecipitation assay (RIPA) buffer (Thermo Fisher Scientific, Rockford, IL, USA) containing a protease and phosphatase inhibitor cocktail (GenDepot, Barker, TX, USA). Protein concentrations were determined using the bicinchoninic acid (BCA) protein assay kit (Thermo Fisher Scientific, Rockford, IL, USA).

Equal amounts of protein lysates (30 μg) were separated on 7.5–10% SDS–PAGE gels and transferred to PVDF membranes at 100 V for 1 h using a wet transfer system (Bio-Rad, Hercules, CA, USA). Membranes were blocked in 5% bovine serum albumin (BSA) prepared in TBS-T (TBS with 0.1% Tween 20) for 2 h at room temperature and then incubated overnight at 4 °C with primary antibodies (1:1500 dilution in TBS-T) under gentle agitation. After washing, membranes were incubated with horseradish peroxidase (HRP)-conjugated secondary antibodies (1:4000 dilution in TBS-T) for 2 h at room temperature. Protein bands were visualized using an enhanced chemiluminescence (ECL) detection reagent (SuperSignal™ West Pico, Thermo Fisher Scientific) and imaged with the Fusion Solo imaging system (Vilber Lourmat, Collegien, France).

### 4.9. Quantitative Real-Time Polymerase Chain Reaction

qPCR was used to assess the mRNA expression of pro-inflammatory mediators. RAW264.7 cells were incubated for 24 h and then co-treated with LPS (1 μg/mL) and SCE for 6 h. Total RNA was extracted using TRIzol reagent (Invitrogen, Carlsbad, CA, USA) according to the manufacturer’s protocol. Complementary DNA (cDNA) was synthesized from the isolated RNA using the AccuPower RT Premix kit (Bioneer, Daejeon, Republic of Korea) with oligo(dT)_18_ primers (Invitrogen).

The cDNA was amplified using a LightCycler 480 real-time PCR system (Roche, Basel, Switzerland). Each PCR reaction contained 10 μL of SYBR Green master mix (Roche), 8 μL of ultrapure water, 1 pmol/μL of each primer, and 1 μL of cDNA template. The thermal cycling conditions were as follows: initial denaturation at 95 °C for 10 min, followed by 45 cycles of denaturation at 95 °C for 10 s, annealing at 50–60 °C for 20 s, and extension at 72 °C for 20 s. The following mouse primers were used: TNFα forward, 5′-AAGCCTGTAGCC CACGTCGTA-3′, reverse, 5′-GGCACCACTAGTTGGTTGTCTTTG-3′; IL-1β forward, 5′-CTGAACTCAACTGTGAAATGCCA-3′, reverse, 5′-AAAGGTTTGGAAGCAGCCCT-3′; IL-6 forward, 5′-CCACTTCACAAGTCGGAGGCTTA-3′, reverse, 5′-GCAAGTGCATCATCGTTGTTCATAC-3′; β-actin forward, 5′-GCAAGTGCTTCTAGGCGGAC-3′, reverse, 5′-AAGAAAGGGTGTAAAACGCAGC-3′. β-Actin was used as the internal control. Relative gene expression levels were calculated using the ΔCt method, normalized to β-actin. Data were acquired using LightCycler 480 software (Roche Applied Science, Santa Clara, CA, USA).

### 4.10. ELISA Analysis

The levels of pro-inflammatory cytokines (TNF-α, IL-1β, and IL-6) in culture supernatants were measured using Quantikine mouse ELISA kits (R&D Systems, Minneapolis, MN, USA), according to the manufacturer’s instructions. RAW264.7 cells (3 × 10^4^ cells/mL) were seeded in six-well plates and incubated at 37 °C in a humidified 5% CO_2_ atmosphere. After 24 h, cells were co-treated with LPS (1 μg/mL) and various concentrations (5, 10, and 20 μg/mL) of callus extract for an additional 24 h. Culture supernatants were then collected, and cytokine concentrations were quantified. Absorbance was measured at 450 nm using a microplate spectrophotometer.

### 4.11. HPLC Fingerprinting Analysis

Chromatographic analysis of the SCE was performed using an Agilent 1260 Infinity HPLC system (Agilent Technologies, Santa Clara, CA, USA) equipped with a UV diode array detector, degasser, and autosampler. Saponarin standard (ChemFaces, Wuhan, China) and filtered SCE samples were prepared at a concentration of 1 mg/mL in pure methanol. Separation was carried out using an Agilent Eclipse XDB-C18 column (150 × 4.6 mm, 5 μm pore size) at a flow rate of 1 mL/min, a column temperature of 23.5 °C, and a detection wavelength of 258 nm. Gradient elution was performed with mobile phase A (water) and mobile phase B (acetonitrile) under the following conditions: 0–5 min, 95% A; 5–20 min, 95% to 0% A; 20–23 min, 0% to 95% A; 23–25 min, 95% A.

### 4.12. Foaming Ability and Stability Studies

Foam formation and stability were evaluated using a modified Bartsch test [52]. The maximum foam height was used to assess foaming ability. A 0.1% solution of Triton X-100, Tween 20, and sodium dodecyl sulfate (SDS) served as positive controls. Foam decay was monitored for 15 min following peak foam generation by measuring the decrease in foam height in a 50 mL graduated cylinder.

### 4.13. Skin Irritation Test

The skin irritation patch test was conducted by AllLive Clinical Trial Research Center (Yongin, Republic of Korea) in accordance with clinical trial ethical guidelines (IRB approval number: IRB-S2305). The test followed the scoring system established by the International Contact Dermatitis Research Group (ICDRG), which uses a six-grade scale. Thirty healthy volunteers (3 males and 27 females) without clinical conditions or medications that could influence the results participated in this study. The average age of the volunteers was 45.77 years. Dried *S. officinalis* callus scrub was diluted in sterile water at a concentration of 1% (20 µL). The test was conducted on a non-lesional area of the subject’s back, which was first cleansed with 70% ethanol and allowed to dry. The test substance was then applied via a patch and left in place for 24 h. After removal of the patch, the test area was marked with a medical skin marker and, following a 1 h interval, the markings were removed. Visual assessments were conducted by a dermatologist at 1 h and 24 h after patch removal. Irritation severity was classified according to ICDRG criteria (Table 1), and mean reaction scores were calculated using the standard skin patch test evaluation chart.

### 4.14. Exfoliation Test

The test of the exfoliation efficacy of the dried *Saponaria officinalis* L. callus scrub was performed by AllLive Clinical Trial Research Center (Yongin, Republic of Korea), in compliance with clinical trial ethics (IRB approval number: IRB-E2305-006). A total of 21 healthy volunteers (1 male and 20 females) with no relevant disease history or medications participated. The average age of the volunteers was 47.57 years. The volar forearm was selected for testing due to its low hair density and consistent skin surface. The skin was allowed to stabilize for at least 30 min in a controlled environment (temperature: 22 ± 2 °C; humidity: 50 ± 10% RH) without airflow or direct sunlight.

Visioscan VC20 plus (Courage + Khazaka electronic GmbH, Bilbao, Spain) was used to capture images before and after scrub application. The desquamation index (DI), indicating keratin shedding from the epidermis, was calculated based on image analysis. Additionally, participants completed a self-assessment questionnaire regarding exfoliation efficacy and product perception.

### 4.15. Statistical Analysis

All data were analyzed using GraphPad Prism version 5.0 (GraphPad Software, La Jolla, CA, USA). One-way analysis of variance (ANOVA) followed by Tukey’s multiple comparison test was used to determine statistical significance. Results are presented as mean ± standard deviation (SD), and *p* < 0.05 was considered statistically significant.

## 5. Conclusions

The callus extract of *Saponaria officinalis* L. (SCE), enriched with saponarin as a bioactive compound, exhibited significant anti-inflammatory effects in LPS-stimulated RAW264.7 cells. In clinical evaluations, the SC scrub demonstrated significant exfoliating effects without causing skin irritation. These findings suggest that the SC scrub may serve as an eco-friendly and sustainable alternative to microplastic-based exfoliants commonly used in the cosmetic industry. Further studies are warranted to explore its long-term safety, formulation compatibility, and large-scale manufacturing potential.

## Figures and Tables

**Table 1:** Evaluation stages and morphological criteria for primary skin irritation caused by patch testing provided by International Contact Dermatitis Research Group (ICDRG). The Symbol indicates severity of morphologic changes in subject’s skin.

Symbol	Score	Morphology	Assessment
-	0	No reaction	Negative reaction
±	0.5	Faint erythema only	Doubtful reaction
+	1	Erythema, Infiltration, Possibly papules	Weak positive reaction
++	2	Erythema, Infiltration, Papules, Vesicles	Strong positive reaction
+++	3	Intense erythema, Infiltration, Coalescing Vesicles	Extreme positive reaction
*	IR	Various morphologies, e.g., soap effect, bulla, necrosis	Irritant reaction of different types

## Data Availability

All data are contained in this article.

## Supplementary Materials

The following supporting information can be downloaded at: https://www.mdpi.com/article/10.3390/plants14101535/s1. Supplementary Table S1. Volunteer information and patch test results. Supplementary Table S2. Volunteer information and exfoliation test results. Supplementary Table S3. Survey results obtained from volunteers (n = 21) after using Callus Scrub. Supplementary Figure S1. Visioscan images showing dead skin cell reduction before and after using the Callus Scrub in 21 volunteers.

## Abbreviations

The following abbreviations are used in this manuscript:

SCO	Stratum corneum
UV	Ultraviolet
TEWL	Transepidermal water loss
SC	*Saponaria officinalis* callus
SCE	*Saponaria officinalis* callus extract
LPS	Lipopolysaccharide
NO	Nitric oxide
iNOS	Inducible nitric oxide synthase
COX-2	Cyclooxygenase-2
MS	Murashige and Skoog
2,4-D	2,4-dichlorophenoxyacetic acid
DMEM	Dulbecco’s Modified Eagle’s Medium
FBS	Fetal bovine serum
PPFD	Photosynthetic Photon Flux Density
DMSO	Dimethyl sulfoxide
KCLB	Korea Cell Line Bank
OD	Optical density
DPBS	Dulbecco’s phosphate-buffered saline
RIPA	Radioimmunoprecipitation assay
BCA	Bicinchoninic acid
BSA	Bovine serum albumin
HRP	Horseradish peroxidase
SDS	Sodium dodecyl sulfate
ICDRG	International Contact Dermatitis Research Group
DI	Desquamation index
ANOVA	One-way analysis of variance
SD	Standard deviation
IRB	Institutional Review Board
HPLC	High-performance liquid chromatography
