# Supplementary material for: Evaluation of Saponin-Rich Callus from Saponaria officinalis L. as a Novel Scrub Material with Significant Exfoliating and Anti-Inflammatory Effects
Source: Plants (Basel). 2025 May 20;14(10):1535. doi: 10.3390/plants14101535 (PMC12115091; doi:10.3390/plants14101535)
Supplement: Supplementary file 1 [file plants-14-01535-s001.zip › plants-3615020-supplementary.pdf]

Supplementary Materials

No.	Sub No.	Gender	Age	Time after removing the patch	
				1 hour	24 hours
1	S2305-C02P01-P01	Male	50	-	-
2	S2305-C02P01-P02	Female	50	-	-
3	S2305-C02P01-P03	Female	50	-	-
4	S2305-C02P01-P04	Female	52	-	-
5	S2305-C02P01-P05	Female	52	-	-
6	S2305-C02P01-P06	Female	51	-	-
7	S2305-C02P01-P07	Female	38	-	-
8	S2305-C02P01-P08	Female	58	-	-
9	S2305-C02P01-P09	Female	37	-	-
10	S2305-C02P01-P10	Female	55	-	-
11	S2305-C02P01-P11	Female	47	-	-
12	S2305-C02P01-P12	Female	55	-	-
13	S2305-C02P01-P13	Male	35	-	-
14	S2305-C02P01-P14	Female	56	-	-
15	S2305-C02P01-P15	Female	39	-	-
16	S2305-C02P01-P16	Female	53	±	-
17	S2305-C02P01-P17	Female	52	-	-
18	S2305-C02P01-P18	Female	32	-	-

19	S2305-C02P01-P19	Female	40	-	-	
20	S2305-C02P01-P20	Female	37	-	-	
21	S2305-C02P01-P21	Male	28	-	-	
22	S2305-C02P01-P22	Female	52	-	-	
23	S2305-C02P01-P23	Female	56	-	-	
24	S2305-C02P01-P24	Female	52	-	-	
25	S2305-C02P01-P25	Female	59	-	-	
26	S2305-C02P01-P26	Female	49	-	-	
27	S2305-C02P01-P27	Female	32	-	-	
28	S2305-C02P01-P28	Female	51	-	-	
29	S2305-C02P01-P29	Female	31	-	-	
30	S2305-C02P01-P30	Female	24	-	-	
	**Total Score**			**0.5**	-	
	**Mean Score**			**0.28**		
	**Grade**			**Grade 1 (Non-irritant)**		

Supplementary Table S1. Volunteer information and patch test results.

Volunteer gender and age and their skin irritation assessment 1 hour and 24 h after patch removal.

No.	Sub No.	Gender	Age	Desquamation index(%)	
				Before	After
1	E2305-006-P001	Female	53	56.02	33.97
2	E2305-006-P002	Female	29	42.36	21.94
3	E2305-006-P003	Female	57	41.8	17.98
4	E2305-006-P004	Female	53	46.32	23.14
5	E2305-006-P005	Female	52	73.14	34.86
6	E2305-006-P006	Female	50	58.71	30
7	E2305-006-P007	Female	58	49.16	31.33
8	E2305-006-P008	Female	39	40.28	22.72
9	E2305-006-P009	Female	49	34.46	17.86
10	E2305-006-P010	Female	51	60.8	35.6
11	E2305-006-P011	Female	52	70.16	49.46
12	E2305-006-P012	Female	59	63.64	37.73
13	E2305-006-P013	Female	52	61.61	38.37
14	E2305-006-P014	Female	51	75.81	47.34
15	E2305-006-P015	Female	52	50.28	29.72
16	E2305-006-P016	Female	47	57.81	29.93
17	E2305-006-P017	Female	51	67.33	41.26
18	E2305-006-P018	Female	42	43.9	20.39
19	E2305-006-P019	Female	48	41.91	23.01

20	E2305-006-P020	Female	31	35.26	18.81
21	E2305-006-P021	Male	23	61.56	36.06
	Average			**53.92**	**30.55**
	Standard Deviation			12.5	9.39
	p-value			-	**<0.0001*** **
	Rate of change			-	**44.07%**

Supplementary Table S 2. Volunteer information and exfoliation test results. Volunteer information of gender and age. Desquamation index (%) showing changes of skin corneocytes before and after using the Callus Scrub in 21 volunteers.

No.	Survey Evaluation Questions	Research participants (n = 21)					
		Very Dissatisfied (Negative)	Dissatisfied	Mostly dissatisfied	Mostly satisfied	Satisfied	Very Satisfied (Positive)
1	The product makes my skin smooth	0	0	0	3	7	11
2	The product makes my skin soft	0	0	0	3	7	11
3	The product helps reduce dead skin cells	0	0	0	3	5	13
4	The product does not irritate the skin	0	0	0	1	7	13
5	Would you buy the product?	0	0	0	2	8	11
6	The product does not irritate the skin	0	0	0	2	8	11

Supplementary Table S3. Survey results obtained from volunteers (n=21) after using Callus Scrub. Numbers correspond to counts of positively responded subjects

Supplementary Figures

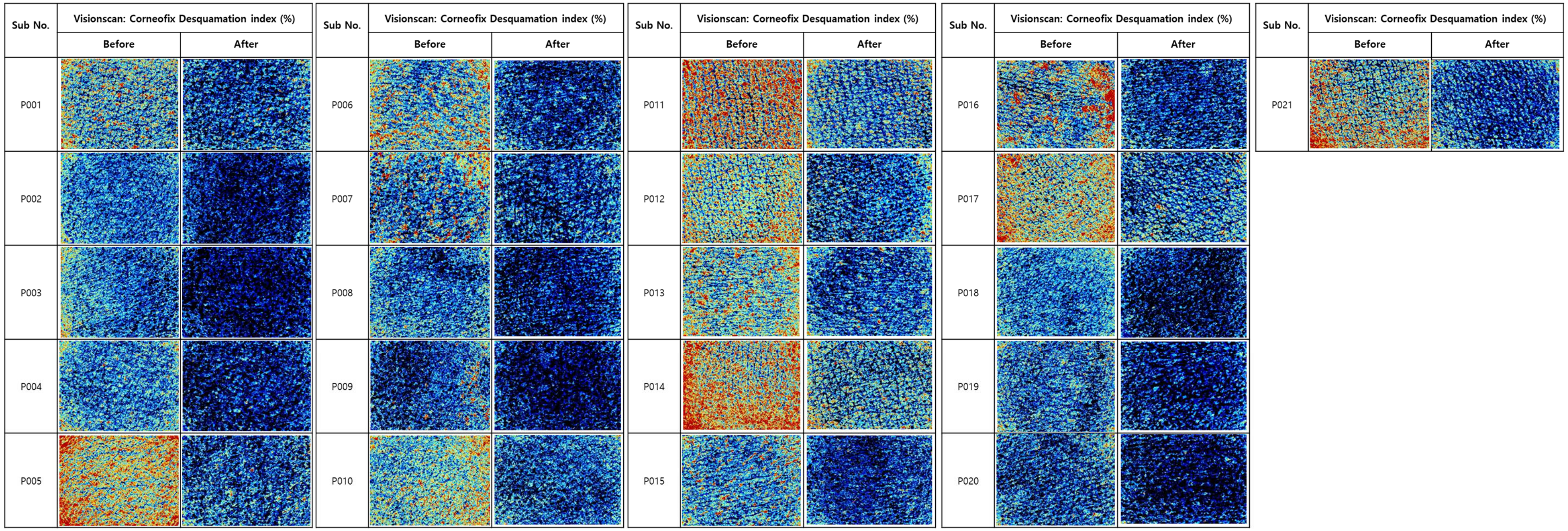

Supplementary Figure S1. Visioscan images showing dead skin cell reduction before and after using the Callus Scrub in 21 volunteers.
